# Development of an indicator framework for assessing nursing quality in interventional therapy for intracranial aneurysms in China

**DOI:** 10.3389/fneur.2024.1403637

**Published:** 2024-12-05

**Authors:** Hai-xia Yang, Ben-fang Fan, Jia Zhao, Jian-hong Ji, Wen-bin Ding, Wei-guang Shen

**Affiliations:** ^1^Department of Radiotherapy, Second Affiliated Hospital of Nantong University, Nantong, Jiangsu, China; ^2^Department of Interventional Medicine, Second Affiliated Hospital of Nantong University, Nantong, Jiangsu, China; ^3^Critical Care Medicine, Second Affiliated Hospital of Nantong University, Nantong, Jiangsu, China

**Keywords:** Delphi method, intracranial aneurysm (IA), interventional therapy nursing, quality assessment, China

## Abstract

**Objective:**

The objective of this study is to devise an indicator system to assess the quality of nursing care in the context of interventional therapy for intracranial aneurysms (IA) in China. This will furnish a standardized and quantitative framework for the monitoring and assessment of nursing quality within the IA interventional therapeutic field.

**Methods:**

The indicators and their associated weights within the evaluation system for nursing quality in interventional therapy for IA were determined based on the theoretical framework of the three-dimensional quality model, specifically the “structure-process-outcome” paradigm. This was achieved by using several methodological approaches, such as literature analysis, semi-structured interviews, expert consultations, the Delphi method, and the analytic hierarchy process.

**Results:**

Expert consultations were conducted over two rounds, with questionnaires distributed via email and WeChat. Both rounds yielded a questionnaire return rate of 100%. Across these consultations, pertinent statistical measures were obtained, such as the expert authority coefficient (Cr), the coefficient of variation (CV), and Kendall’s harmony coefficient, which exhibited values of 0.886 and 0.952, 0–0.193 and 0–0.185, and 0.138 and 0.149, respectively. These findings indicated statistically significant differences (*p* < 0.01). Notably, the indicators within the final iteration of the evaluation system for nursing quality in interventional therapy for IA are categorized into 3 tiers: primary indicators, encompassing 3 metrics; secondary indicators, comprising 10 metrics; and tertiary indicators, consisting of 36 indicators.

**Conclusion:**

The indicator system devised for assessing nursing quality in interventional therapy for IA, as outlined in this study, possesses a high level of scientific rigor and reliability in China. It aptly captures the unique nuances inherent in IA management during interventional therapy nursing, thereby serving as a valuable reference point for the assessment of nursing quality within the context of IA interventional therapy.

## 1 Introduction

An intracranial aneurysm (IA) denotes a localized pathological expansion of the arterial wall within the cranium. Globally, the incidence rate of IA varies between 1 and 6% (1). Based on whether they have ruptured or not, intracranial aneurysms can be classified as ruptured intracranial aneurysms (RIA) and unruptured intracranial aneurysms (UIA). Most patients with UIA often have no clinical symptoms and are difficult to detect. However, the rupture of an IA is sudden and poses a significant life-threatening risk to patients (2). Once an IA ruptures, patients may experience sudden, severe headaches (about 70% of patients experience thunderclap headaches), pain in the back of the neck, and may develop altered consciousness or even coma within a short period. In severe cases, it can be life-threatening (3). The rupture of an IA frequently leads to an aneurysmal subarachnoid hemorrhage, often accompanied by a poor prognosis, thereby delineating IA as a critical cerebrovascular disorder with significant implications for human health (4). Based on available literature, the mortality rate subsequent to IA rupture can escalate to as high as 50% (5, 6). Therefore, early treatment and intervention for intracranial aneurysms are crucial methods for improving patient outcomes.

Treatment of intracranial aneurysms (IA) includes surgical craniotomy and endovascular intervention (7, 8). With advancements in neurointerventional technology, interventional therapy has emerged as the preferred treatment for IA, significantly reducing the rebleeding and mortality rates associated with ruptured aneurysms (9). However, complications associated with endovascular treatment of intracranial aneurysms should not be overlooked. Common complications include aneurysm re-rupture, cerebral vasospasm, delayed cerebral ischemia, cerebral thrombosis or embolism, hydrocephalus, epilepsy, and infection. Patients may experience coma, aphasia, hemiparesis, or even death. If medical and nursing staff do not promptly detect and properly manage these issues, the patient’s safety and health may be significantly threatened. Therefore, specialized monitoring and care are essential following endovascular embolization treatment.

Nurses are the most direct and frequent contact for patients during their treatment and play a crucial role in post-intervention management. Lilin (10) found that implementing proactive nursing care for patients undergoing low-grade Hunt-Hess intracranial aneurysm endovascular embolization reduced the incidence of complications such as aneurysm re-rupture, cerebral vasospasm, and cerebral thrombosis from 35 to 5%. Fangzhen et al. (11) developed preventive nursing intervention plans to reduce perioperative bleeding complications and improve care quality. Juan et al. (12) enhanced the quality of life for patients undergoing endovascular treatment for intracranial aneurysms through thorough preoperative preparation, precise intraoperative coordination, and postoperative rehabilitation, as well as perioperative health education and nursing interventions. Each nursing measure during the intervention period is related to patient safety and prognosis (13). Scientific, standardized nursing practices and early preventive care interventions can reduce postoperative complications and improve patient quality of life (11). Therefore, effectively improving the quality of nursing care during IA intervention is of significant importance.

The outcomes of patients undergoing interventional therapy are profoundly influenced by the quality of nursing care they receive. Ensuring patient safety and treatment efficacy necessitates a systematic and comprehensive assessment of nursing quality, which plays a pivotal role in diminishing incidence rates of complications (14). In the late 1980s, Donabedian proposed the “structure-process-outcome” three-dimensional quality framework, aimed at evaluating healthcare service quality from three dimensions: structural quality, process quality, and outcome quality (15). Structural quality evaluation focuses on assessing the characteristics of nursing system resources, including organizational management, human resources, and material provisions. Process quality evaluation primarily refers to the compliance and implementation of nursing activities and measures by nursing staff. Outcome quality evaluation assesses the results produced by the structure and process, representing the final evaluation of nursing effectiveness, including patient or family satisfaction with nursing services and patient health outcomes (16). The American Nurses Association was the first to apply the “structure-process-outcome” framework to develop a nursing quality evaluation indicator system (15). Subsequently, researchers worldwide have used this model to conduct related studies on nursing quality evaluation indicator systems (17).

Only few studies have addressed the development of assessment indicators pertaining to nursing quality in both unruptured IA interventional procedures and IA surgical interventions (18, 19). However, the indicator constructed is not comprehensive enough in terms of the scope and content of the evaluation criteria. Yuan and Ma (18) developed 21 perioperative nursing evaluation indicators for unruptured intracranial aneurysm interventions using literature analysis and the Delphi method. Their evaluation targets are patients undergoing intervention for unruptured aneurysms, focusing primarily on monitoring postoperative complications. However, their framework lacks structural and outcome indicators, and the coverage is not comprehensive. Additionally, they did not set indicator weights, which makes it difficult to clearly reflect the importance of each indicator. Li et al. (19) developed a nursing-sensitive indicator system for patients with ruptured intracranial aneurysms using evidence-based methods and the Delphi method. This system is aimed at patients undergoing surgical treatment for ruptured intracranial aneurysms in neurosurgery and is suitable for monitoring and managing postoperative nursing quality. However, it is not strongly guiding for nursing care in IA interventional treatment. Hence, this study endeavors to devise an indicator system for evaluating IA interventional therapy nursing, based on the theoretical framework of the three-dimensional quality evaluation model—“structure-process-outcome”—and using the Delphi method along with the analytic hierarchy process in China. Our objective is to furnish a scientifically grounded reference for evaluating nursing quality in interventional therapy for IA.

## 2 Study method

### 2.1 The establishment of the study team

The team members for this study were selected based on the study objective and their proficiency in IA. A total of seven personnel were ultimately included in the study team. Among them, one physician from the interventional department, serving as the chief physician in the field of neurointerventional diagnosis and treatment, was included. The chief head nurse from the internal medicine department, acting as the chief nurse in interventional therapy nursing, was responsible for the design, quality control, initial review, and supplementation of indicators, as well as the selection and invitation of experts for the entire study. Additionally, a head nurse, functioning as the deputy chief nurse in neurointerventional nursing, was included. Two specialized nurses, serving as supervising nurses in neurointerventional nursing, were responsible for formulating and compiling specific indicator-related items and distributing expert consultation questionnaires. Finally, two nursing graduate students were included, tasked with literature retrieval, data organization, and analysis. This study followed the Declaration of Helsinki and was approved by the Ethics Committee of Second Affiliated Hospital of Nantong University. Written informed consent was obtained from all participants.

### 2.2 Drafting initial indicators

#### 2.2.1 Literature analysis

**Figure 1:**
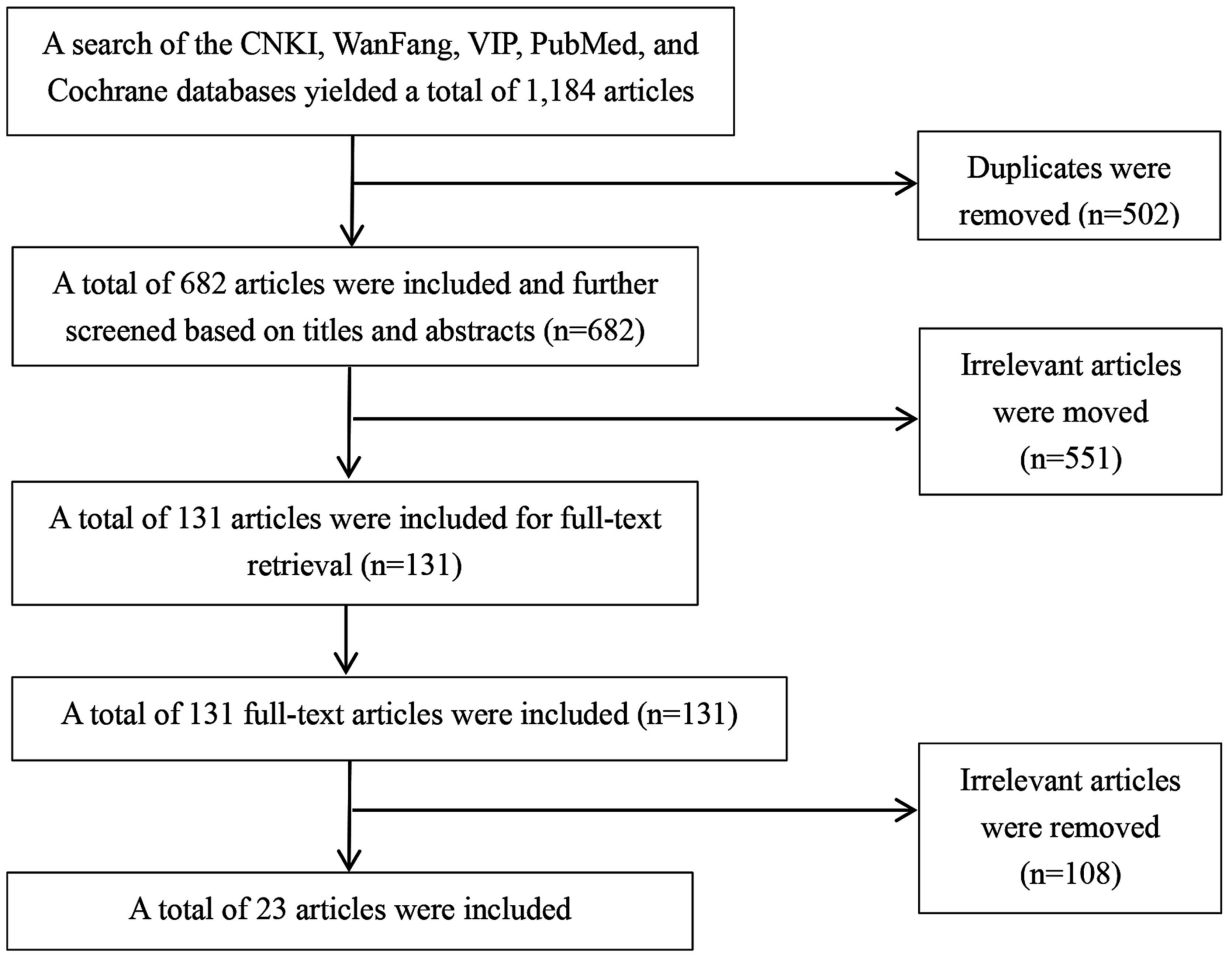
Literature screening process.

Literature retrieval was carried out using various databases, with the inclusion dates of articles set from January 2012 to December 2022. These databases included China National Knowledge Infrastructure (CNKI), Wanfang Data, CQVIP database, PubMed, Cochrane Library, among others. The search terms used were “intracranial aneurysm,” “intervention,” and “nursing,” both in English and Chinese. Two researchers independently conducted the literature retrieval, screening, and quality assessment process. The grading and assessment of literature quality adhered to the evidence grading and quality assessment standards established by the Joanna Briggs Institute (JBI) Center for Evidence-Based Healthcare. In cases of divergent opinions between the researchers, consultation with experts and nurses specializing in IA interventional therapy or interventional therapy nursing was sought. The inclusion criteria for the literature are as follows: ① The study content involves literature related to intracranial aneurysm interventional nursing quality management, quality evaluation, or the effectiveness of nursing interventions; ② The literature types include guidelines, expert consensus, systematic reviews, and original research. Exclusion criteria: ① Conference abstracts, reports, or literature with low quality ratings; ② Literature for which the full text is not available, or duplicate and irrelevant literature. A preliminary search yielded 1,184 articles. After reviewing titles, abstracts, and full texts, and excluding duplicates, unavailable full texts, and irrelevant articles, 23 articles were finally included (7–9, 11–13, 18–34). These included 4 guidelines (7, 8, 28, 29), 3 expert consensus documents (22–24), 3 systematic reviews (25–27), and 13 original research articles (9, 11–13, 18–21, 30–34). The literature screening process is shown in Figure 1.

Based on the literature search results and clinical nursing practice, preliminary evaluation indicators were proposed. This study uses the “three-dimensional quality structure” model as the theoretical basis to establish the framework for the IA interventional nursing quality evaluation indicator system. Structural quality, process quality, and outcome quality are defined as primary indicators. Subsequently, secondary and tertiary indicators are developed based on the content of each dimension, in combination with the characteristics of specialized nursing for IA interventions.

#### 2.2.2 Semi-structured interviews

Based on literature analysis, semi-structured interviews were conducted to refine and augment the indicators. By using purposive sampling, interviews were conducted with neurointervention doctors, interventional nurses, and patients (or their families) undergoing IA interventional treatment at a tertiary hospital in Nantong, Jiangsu Province, from February to April 2024. The interviews were terminated when redundant information was consistently presented, and no new themes emerged.

Inclusion criteria for medical personnel to be interviewed comprised of: ① Doctors holding a master’s degree or above, possessing a minimum of 5 years of experience in neurointerventional diagnosis and treatment, and nurses holding a bachelor’s degree or above, with at least 5 years of experience in interventional therapy nursing. ② Both doctors and nurses were required to hold intermediate or higher professional titles; ③ Both doctors and nurses possessed extensive experience in the neurointerventional domain and demonstrated proficient communication skills. Exclusion criteria included: pregnancy, lactation, or absence from work for at least 3 months during the year of participant selection for the study. The interview outline encompassed the following aspects: (1) What are your primary concerns and apprehensions when treating or providing care for patients with IA? (2) What factors do you believe influence the quality of interventional therapy nursing in IA? (3) How do you propose the enhancement of the quality of interventional therapy nursing in IA? (4) From your perspective, which facets can be used to assess the quality of interventional therapy nursing in IA?

Inclusion criteria for patients (or their family members) eligible for interviews encompassed: ① patients undergoing IA interventional therapy within the ward or their respective family members; ② expressed willingness to participate in the study and demonstrated proficient expression and communication abilities. Exclusion criteria for patients (or their family members) to be interviewed included participants who withdrew from the interview process. The interview outline comprised the following inquiries: (1) What are your (or your family’s) primary concerns during the interventional treatment? (2) What difficulties have you encountered during the treatment and nursing process? (3) How have you addressed these issues? (4) What kind of assistance would you like to receive to better help you face and resolve these challenges?

Before the interviews, appointments were scheduled with the interviewees to confirm the time. The purpose and significance of the interview were explained to them, and confidentiality of the interview content was assured. The interviews were conducted in a quiet environment, such as a hospital ward or teaching room, with each interview lasting 15–30 min. After interviewing 7 doctors, 9 nurses, and 8 patients, information saturation was reached. Additional interviews with 2 more participants yielded no new content, leading to the conclusion of the interview process.

The interview recordings were transcribed into text format within a 24-h timeframe following the interviews. Subsequently, the transcribed text underwent organization, analysis, and summarization using Colaizzi’s seven-step method. This method facilitated the extraction of themes pertinent to the evaluation of nursing quality in IA interventional therapy, including aspects such as “nursing personnel allocation,” “comprehensive and dynamic evaluation of specialized nursing,” “identification and prevention of complications,” “awareness of intervention procedures,” and “implementation of patient health education.”

#### 2.2.3 Group meeting discussion

Based on the findings from literature analysis and semi-structured interviews, the study team members proceeded to draft indicators for evaluating nursing quality in IA interventional therapy. Subsequently, a conference group consisting of five experts in interventional therapy nursing was convened. This group included one head nurse from the internal medicine department, two head nurses from the neurointerventional ward department, and two provincial-level specialist nurses. Their task was to engage in discussions and analyses, and to modify the indicators. As a result, preliminary indicators for evaluating nursing quality in IA interventional therapy were developed. These indicators comprised 3 primary indicators, 10 secondary indicators, and 46 tertiary indicators. Each tertiary indicator was detailed to include the indicator name, calculation formula, and the method used for data collection.

### 2.3 Drafting the questionnaire for consultation

The questionnaire encompasses the following sections: (1) Introduction: this section provides an overview of the background, purpose, significance, and instructions for completing the questionnaire. (2) Indicator Consultation Table: in this section, a table is provided listing the indicators for evaluating the quality of nursing care for IA interventional therapy. Experts were required to score the importance of each indicator using a five-point Likert scale. Additionally, they were encouraged to provide comments after each indicator. (3) Expert Data: this section collects general information about the experts, including age, education level, professional title, specialized field, and years of experience in the field. Moreover, it includes a survey on the authority level of the experts in their respective fields, covering their proficiency on the consulted items and the basis of their judgments.

### 2.4 The selection of experts to be consulted

Inclusion criteria for consultation experts were as follows: (1) Education: Bachelor’s degree or higher. Professional title: intermediate or higher. (2) Nurses with a minimum of 10 years of experience in neurointerventional therapy nursing or interventional therapy nursing management at tertiary grade A hospitals in China, with extensive experience in interventional clinical nursing. They were required to possess extensive clinical nursing experience and critical thinking abilities. (3) Physicians holding the position of deputy chief physician or higher, with a minimum of 10 years’ experience in neurointerventional diagnosis and treatment at tertiary grade A hospitals. (4) Demonstrated enthusiasm for participation in the study and voluntary engagement.

### 2.5 Consulting the experts

Questionnaires were distributed and collected via WeChat and email platforms. Experts were instructed to return the completed questionnaires within a 7-day period from the date of distribution. Following the initial round of questionnaire dissemination and retrieval, the study team analyzed the opinions and feedback provided by the experts. Based on their input, indicators were selected, modified, or supplemented as deemed necessary. The revised set of indicators underwent a second round of expert consultation. Upon completion of the two rounds of expert consultations, a consensus among the experts was observed, marking the conclusion of the consultation process. Criteria for screening indicators included: importance rating > 3.5 points. Full score rate > 20%. Coefficient of variation (CV) < 0.25 (35). To mitigate memory bias, a 20-day interval was implemented between the two rounds of expert consultation.

### 2.6 Statistical methods

Data in this study were analyzed and processed using SPSS 20.0 and yaahp 10.0 software packages. Measurement and enumeration data were characterized using mean, standard deviation, frequency, and percentage. The level of expert participation, authority, and opinion concordance were evaluated through metrics such as questionnaire return rates, rates of opinion submission, expert Cr, CV, and Kendall’s harmony coefficient (W). The determination of indicator weights was conducted using the analytic hierarchy process. Judgment matrices were formulated to rank indicators, assess their consistency, and derive the weights of individual indicators as well as the combined weights of indicator sets.

## 3 Results

### 3.1 General information of the experts

Following two rounds of expert consultations, this study ultimately enlisted the participation of 25 experts from 10 tertiary grade-A hospitals situated across Jiangsu Province, Zhejiang Province, Shanghai City, and Beijing City. A summary of the general information pertaining to these experts is presented in Table 1.

**Table 1:** General data about the experts (*n* = 25).

Item	Group	Number	Proportion (%)
Age	20–35	4	16
	36–50	13	52
	>50	8	32
Years of working experience	10–15	6	24
	16–20	7	28
	>20	12	48
Gender	Male	3	12
	Female	22	88
Professional title	Intermediate level	6	24
	Deputy senior	11	44
	Advanced level	8	32
Education level	Bachelor degree	17	68
	Master’s degree	6	24
	Doctor of Medicine	2	8
Specialized field	Interventional nursing	20	80
	Interventional medicine	4	16
	Nursing education	1	4

### 3.2 Level of expert participation and authority

For both rounds of expert consultation, a total of 25 questionnaires were distributed and subsequently returned, resulting in a 100% effective questionnaire return rate for both rounds. The rates of expert opinion submissions were 16 out of 25 (64%) for the first round and 4 out of 25 (16%) for the second round. Expert Cr values were 0.886 and 0.952, respectively, for the two rounds, indicating a high level of authority among the experts.

### 3.3 Level of expert opinion consistency

The CV for the two rounds of expert consultations ranged from 0–0.193 and 0–0.185, respectively. Additionally, the Kendall’s harmony coefficient was 0.138 and 0.149, respectively, for the two rounds, and the difference was statistically significant (*p* < 0.01). These findings indicate a high level of expert concordance and a good consistency of opinions among the experts.

### 3.4 Results of expert consultations

The indicators underwent modifications over two rounds based on indicator screening criteria, expert opinions, and discussions within the study team. The specific revisions made during the first round of indicator modification are outlined below: (1) twelve tertiary indicators were removed, including those pertaining to the occurrence rates of phlebitis, nosocomial infection, and nursing-related adverse events. These were deemed as general indicators that did not specifically reflect the distinctive characteristics of IA. Additionally, “the accuracy rate of assessment of patient anxiety and depression” was eliminated due to the requirement of specialized scales and low patient compliance in critical conditions. (2) Ten tertiary indicators were modified: The four indicators—“basic theories and knowledge of IA, operational skills, identification and handling of common complications, and pass rate of assessment for emergency tackling capability”—were consolidated into “the implementation rate of training program for interventional therapy nursing in IA, coverage rate of the training content, and the pass rate of assessment of nurse training mastery.” Furthermore, indicators such as “body position management for patients with IA, target blood pressure management, and pass rate of volume management” were modified to reflect their respective “standard implementation rates” as a more accurate reflection of completion. Additionally, “the rate of timely treatment of abnormality in special laboratory indicators” was revised to “the implementation rate of standardized treatment of abnormal laboratory indicators” for enhanced accuracy. (3) Three tertiary indicators were introduced, including “the percentage of nurses cognizant of intraoperative conditions of the interventional surgery,” with the goal to ensure the awareness of nurses to surgical processes for effective postoperative monitoring. The other two added indicators were “the implementation rate of nursing in accordance with standards for the punctured sites” and “the completion rate of assessment of severity of IA (Hunt-Hess),” both considered distinctive contents of nursing and evaluation for interventional surgery of IA.

During the second round of indicator revision, the following adjustments were made: (1) the tertiary indicator “The incidence rate of complications in local blood vessels in limbs following arterial puncture” was removed due to perceived overlap with “the incidence rate of complications following interventional surgery of IA.” Four tertiary indicators were modified as follows: “The rate of patients (or their family members) cognizant of healthcare points for admission, hospitalization, and discharge” was revised to “The rate of patients (or their family members) cognizant of healthcare points for the prevention of bleeding subsequent to IA rupture and the rate of patients (or their family members) cognizant of healthcare points for discharge and follow-ups.” The objective of this modification was to encompass essential healthcare points regarding IA rupture, bleeding prevention, discharge, and follow-up care. “The incidence rate of complications of IA” was adjusted to “the incidence rate of complications of IA (including rebleeding, rupture, subsequent to cerebrovascular spasm, cerebral ischemia related to interventional therapy, complications of local puncture in artery, etc.)” for more targeted monitoring of complications. The final indicator system for evaluating nursing quality in interventional therapy for IA comprises 3 primary indicators, 10 secondary indicators, and 36 tertiary indicators (see Table 2).

**Table 2:** Indicators for assessing the quality of nursing care in interventional therapy for IA.

Indicators	Full score rate (%)	Score of the importance (points, $\bar{x}$ ± s)	Coefficient of variation (CV)	Weight	Collective weight
Structure indicators	88.0	4.88 ± 0.33	0.068	0.330	0.330
I-1 Human resources	84.0	4.84 ± 0.37	0.077	0.334	0.055
I-1-1 Nurse-to-patient ratio	92.0	4.92 ± 0.28	0.056	0.508	0.028
I-1-2 Composition ratio of the proficiency levels of nurses	80.0	4.76 ± 0.52	0.110	0.492	0.027
I-2 Environmental facilities	68.0	4.64 ± 0.57	0.123	0.320	0.083
I-2-1 Compliance rate of ward environment management	64.0	4.64 ± 0.49	0.106	0.317	0.026
I-2-2 Adequacy rate of emergency medications	100.0	5.00	0.000	0.342	0.028
I-2-3 Adequacy rate of specialized instruments/equipment	100.0	5.00	0.000	0.342	0.028
I-3 Education and training	100.0	5.00	0.000	0.345	0.118
I-3-1 Implementation rate of training program for interventional therapy nursing in IA	92.0	4.92 ± 0.28	0.056	0.330	0.028
I-3-2 Coverage rate of training content for interventional therapy nursing in IA	100.0	5.00	0.000	0.335	0.028
I-3-3 Pass rate of nurse training assessment	100.0	5.00	0.000	0.335	0.028
II Process indicators	100.0	5.00	0.000	0.338	0.338
II-1 Specialized nursing assessment	100.0	5.00	0.000	0.201	0.169
II-1-1 Accuracy rate of consciousness assessment [Glasgow Coma Scale (GCS) Score]	100.0	5.00	0.000	0.167	0.028
II-1-2 Accuracy of pupil assessment	100.0	5.00	0.000	0.167	0.028
II-1-3 Accuracy rate of vital sign monitoring	96.0	4.96 ± 0.20	0.040	0.166	0.028
II-1-4 Accuracy rate of muscle strength assessment	96.0	4.96 ± 0.20	0.040	0.166	0.028
II-1-5 Completion rate of IA severity (Hunt-Hess) assessment	100.0	4.96 ± 0.20	0.040	0.166	0.028
II-1-6 Completion rate of focal neurological dysfunction assessment (Language, swallowing, vision, etc.)	100.0	5.00	0.000	0.167	0.028
II-2 Specialized nursing interventions	100.0	5.00	0.000	0.201	0.280
II-2-1 Implementation rate of position management standards for IA patients	92.0	4.92 ± 0.28	0.056	0.099	0.028
II-2-2 Implementation rate of standardized airway safety management measures	100.0	5.00	0.000	0.101	0.028
II-2-3 Implementation rate of target blood pressure management standards	100.0	5.00	0.000	0.101	0.028
II-2-4 Implementation rate of volume management standards	92.0	4.92 ± 0.28	0.056	0.099	0.028
II-2-5 Implementation rate of special medication usage standards	96.0	4.96 ± 0.20	0.040	0.100	0.028
II-2-6 Implementation rate of preoperative preparation standards for interventional therapy for IA	100.0	5.00	0.000	0.101	0.028
II-2-7 Implementation rate of standardized nursing care measures in puncture sites	100.0	5.00	0.000	0.101	0.028
II-2-8 Implementation rate of hydration therapy standards after contrast agent use	88.0	4.88 ± 0.33	0.068	0.099	0.028
II-2-9 Implementation rate of standardized catheter care measures	96.0	4.92 ± 0.40	0.081	0.099	0.028
II-2-10 Implementation rate of standardized handling measures for abnormal laboratory indicator (blood biochemistry, blood coagulation, etc.)	92.0	4.88 ± 0.44	0.090	0.099	0.028
II-3 Nursing of common symptoms and signs	96.0	4.96 ± 0.20	0.040	0.199	0.139
II-3-1 Implementation rate of nursing standards for pain (headache, posterior neck pain)	96.0	4.96 ± 0.20	0.040	0.202	0.028
II-3-2 Implementation rate of nursing standards for vomiting/aspiration	96.0	4.96 ± 0.20	0.040	0.202	0.028
II-3-3 Implementation rate of standardized nursing measures for preventing constipation	84.0	4.84 ± 0.37	0.077	0.197	0.027
II-3-4 Implementation rate of standardized temperature control measures for fever patients	92.0	4.92 ± 0.28	0.056	0.200	0.028
II-3-5 Implementation rate of nursing measures for limb function exercise	88.0	4.88 ± 0.33	0.068	0.199	0.028
II-4 Observation and management of complications	100.0	5.00	0.000	0.201	0.056
II-4-1 Implementation rate of standardized preventive measures for complications in patients with IA (rebleeding subsequent to rupture, cerebrovascular spasm, cerebral ischemia related to interventional therapy, etc.)	96.0	4.96 ± 0.20	0.040	0.498	0.028
II-4-2 Implementation rate of standardized prevention measures for venous thromboembolism (VTE)	100.0	5.00	0.000	0.502	0.028
II-5 Health education	92.0	4.92 ± 0.28	0.056	0.198	0.089
II-5-1 Rate of patients aware of healthcare points relevant to the prevention of rupture and bleeding of IA	100.0	5.00	0.000	0.508	0.028
II-5-2 Rate of patients aware of healthcare points for discharge and follow-ups	84.0	4.84 ± 0.37	0.077	0.492	0.027
III Outcome indicators	92.0	4.92 ± 0.28	0.056	0.332	0.332
III-1 Patient outcome indicators	92.0	4.92 ± 0.28	0.056	0.506	0.053
III-1-1 Incidence rate of complications associated with IA (such as rebleeding subsequent to rupture, cerebrovascular spasm, cerebral ischemia related to interventional therapy, and complications of local puncture in artery)	88.0	4.88 ± 0.33	0.068	0.524	0.028
III-1-2 Average length of hospital stay	60.0	4.44 ± 0.82	0.185	0.476	0.025
III-2 Patient satisfaction rate	80.0	4.8 ± 0.41	0.085	0.494	0.026
III-2-1 Satisfaction rate of patients (or their family members) with nursing care	76.0	4.68 ± 0.69	0.148	1.000	0.026

## 4 Discussion

This study analyzes the included literature and combines it with clinical practice to derive practical and scientific evaluation indicators for IA interventional treatment nursing quality in China. These indicators mainly include: nursing assessment (consciousness, pupil reaction, vital signs monitoring, muscle strength, Hunt-Hess score, language, swallowing, vision, etc.), specialized nursing (positioning, target blood pressure, fluid management, body temperature, catheter management, special medication administration, etc.), common symptom care (headache, vomiting, aspiration, constipation, limb dysfunction, etc.), medical quality (re-bleeding of intracranial aneurysm, cerebral vasospasm, treatment-related cerebral ischemia, localized vascular hematoma at the puncture site, pseudoaneurysms, etc.), and environmental facilities (quiet environment, availability of emergency facilities). The indicators extracted through literature analysis have a scientific evidence base and professional theoretical support.

Based on the literature analysis, semi-structured interviews were conducted with doctors, nurses, and patients (or their families). Through these interviews, key themes related to IA interventional nursing quality evaluation were identified, including “nursing staff allocation,” “comprehensive and dynamic specialized nursing assessment,” “complication identification and prevention,” “awareness of the interventional procedure,” “implementation of patient health education,” “policies and protocols,” and “training indicators.” These themes were used to validate the research indicators obtained through the literature review. Additionally, a panel of IA interventional treatment nursing experts was organized to further discuss, analyze, and revise the scientific validity, sensitivity, representativeness, independence, and feasibility of the indicators, leading to further refinement and enhancement of the indicator content.

The selection of consultation experts is integral to the Delphi method consultation process (36). In this study, a total of 25 experts were ultimately enlisted, specializing in clinical interventional therapy, interventional therapy nursing, nursing management, or nursing education. Notably, 8 experts (32%) held a master’s degree or higher, while 19 (76%) held a deputy chief title or above. All 25 experts (100%) have a working experience of >10 years in their respective fields, indicating a robust theoretical knowledge base and extensive practical expertise relevant to the focus of the study. The questionnaire return rate for both rounds of expert consultation stood at 100%, reflecting a high level of engagement and enthusiasm among the experts. Additionally, the submission rates for proposed revisions by experts were 64 and 16%, respectively. Expert authority, as denoted by the expert Cr, was deemed significant. A Cr value ≥0.7 indicates acceptable reliability, while a value ≥0.8 signifies a strong command of the content (37, 38). In this study, the expert Cr for the two rounds of consultation was 0.886 and 0.952, respectively, indicating a high level of expert authority. Concordance among experts was evaluated using the CV and the Kendall’s harmony coefficient (W) (39). The CV ranged from 0 to 0.193 and from 0 to 0.185 for the two rounds of expert consultation, respectively. Additionally, the Kendall’s harmony coefficients were calculated as 0.138 and 0.149, respectively (*p* < 0.01), indicating a high level of agreement among experts and consistent expert opinions. Furthermore, the importance weights of each indicator were determined using the analytic hierarchy process, ensuring appropriate weighting of each indicator. Consequently, the developed indicator system for evaluating nursing quality in interventional therapy for IA is deemed highly scientific and reliable.

The indicator system developed in this study for evaluating nursing quality in interventional therapy for IA comprehensively addresses the entire perioperative period of IA interventional therapy nursing in China. These indicators emphasize fundamental aspects of nursing care during IA interventional therapy and are seamlessly integrated throughout the structure-process-outcome indicator system.

Among the structural indicators, education and training (a secondary indicator) carry the highest weight (0.345). This prominence can be attributed to the severity of IA conditions of the patients, the specialized nature of the surgery, and the considerable risks associated with the perioperative period. Providing disease-specific educational training to nurses enhances their clinical acumen and risk mitigation abilities, thereby ensuring the quality of nursing care provided. This finding is consistent with the research findings of Li et al. (19). Additionally, the composite adequacy rate of emergency rescue drugs (a tertiary indicator) holds significant weight in total (0.028). This is attributable to the essential role of specialized drugs such as nimodipine, mannitol, antihypertensives, and sedatives in emergency situations involving IA patients. The adequacy of these drugs is imperative for the successful rescue and management of patients with IA.

Among the process indicators (secondary indicators), the evaluation of specialized nursing, treatment of specialized nursing, and observation and management of complications collectively carry a relatively high weight (0.201). This underscores the pivotal role of these three indicators in interventional therapy nursing for IA. The amalgamation of these secondary indicators comprises of 18 tertiary indicators, collectively contributing to a substantial combined weight (0.028), with each indicator playing a critical role. Blood pressure management emerges as a focal point and challenge in the perioperative care of patients with IA (40). Currently, no standardized consensus exists regarding the optimal target blood pressure range and selection of antihypertensive medications for patients with IA. Some studies indicate maintaining a systolic blood pressure < 160 mmHg before initiating IA therapy, with post-therapy blood pressure control targets are tailored to the baseline levels of patients to prevent inadequate cerebral perfusion caused by hypotension (22). The implementation rates of preventive measures for complications of IA (including rebleeding subsequent to rupture, cerebrovascular spasm, and cerebral ischemia) and venous thromboembolism (VTE), both tertiary indicators, collectively hold significant weight (0.028). This underscores the pivotal role of complication prevention in quality management procedures. Proactive nursing care has been shown to effectively reduce complication occurrence rates associated with IA and correlate with enhanced patient prognosis (14). Hence, enhancing the proactive nursing care abilities of nurses to anticipate complications is imperative for enhancing the quality of nursing care following IA interventional surgery. The monitoring and implementation of these indicators and preventive measures are intricately linked to nursing responsibilities, highlighting the unique characteristics of interventional therapy nursing for IA.

Enhancing patient outcomes is the paramount objective of nursing care quality (41). Among the outcome indicators, the collective contribution of the incidence rates of IA complications (tertiary indicators) holds the highest weight (0.028). This alignment with fundamental indicators used to assess nursing interventions is noteworthy. Research demonstrates the pivotal role nurses play in enhancing patient outcomes, with nursing interventions shown to reduce postoperative complication rates among patients with IA from 35 to 5% (10). Therefore, using the incidence rate of IA complications as a metric for evaluating nursing quality is deemed scientifically robust and reliable.

In addition, this study has structured the indicator system around three core dimensions: structure, process, and outcome. Further refinement was achieved by incorporating specific considerations of interventional therapy nursing for IA during the perioperative period. Primarily, within this indicator system, each item is intricately linked to the clinical nursing practice of interventional therapy nursing for IA. This approach enables nurses to efficiently implement interventions by addressing individual indicators, thereby providing crucial clinical guidance. Secondly, each tertiary indicator encompasses indicator name, calculation formula, and data collection methods. Nursing managers can leverage these indicator items to assess both the quality of nursing care in interventional therapy for IA and the professional competencies of nurses, both horizontally and vertically. Consequently, this indicator system is highly practical and facilitates convenient quantification. This indicator system has good practicality and measurability in China.

However, there was some limitations in the present study. The indicators developed are from the consultation from experts, which could be biased. It is worth noting that this study is somewhat constrained by its lack of corresponding clinical application thus far. Our subsequent steps entail conducting tests to assess the reliability and validity of the indicators, followed by their validation in clinical practice.

## 5 Conclusion

The IA interventional treatment nursing quality evaluation indicator system developed in this study includes 3 primary indicators, 10 secondary indicators, and 36 tertiary indicators. The outcomes of this endeavor are characterized by scientific rigor, reliability, and practical applicability, providing a valuable reference for nursing quality evaluation in IA interventional therapy.

## Data Availability

The original contributions presented in the study are included in the article/supplementary material, further inquiries can be directed to the corresponding author.
